# Expanded mutational spectrum of the *GLI3* gene substantiates genotype–phenotype correlations

**DOI:** 10.1007/s13353-012-0109-x

**Published:** 2012-08-18

**Authors:** Aleksander Jamsheer, Anna Sowińska, Tomasz Trzeciak, Małgorzata Jamsheer-Bratkowska, Anita Geppert, Anna Latos-Bieleńska

**Affiliations:** 1 Department of Medical Genetics, University of Medical Sciences, ul. Grunwaldzka 55 paw. 15, 60-352 Poznań, Poland; 2 NZOZ Center for Medical Genetics GENESIS, ul. Grudzieniec 4, 60-601 Poznań, Poland; 3 Department of Orthopedics and Traumatology, University of Medical Sciences, ul. 28 czerwca 1956 r. 135/147, 61-545 Poznań, Poland; 4 Department of Environmental Hygiene, National Institute of Public Health—National Institute of Hygiene, 24 Chocimska Street, 00-791 Warsaw, Poland; 5 Department of Neurology, University of Medical Sciences, ul. Przybyszewskiego 49, 60-355 Poznań, Poland

**Keywords:** Greig cephalopolysyndactyly, GCPS, Preaxial polydactyly type IV, PPD-IV, Genotype–phenotype correlation, *GLI3*

## Abstract

Greig cephalopolysyndactyly syndrome (GCPS) and isolated preaxial polydactyly type IV (PPD-IV) are rare autosomal dominant disorders, both caused by mutations in the *GLI3* gene. GCPS is mainly characterised by craniofacial abnormalities (macrocephaly/prominent forehead, hypertelorism) and limb malformations, such as PPD-IV, syndactyly and postaxial polydactyly type A or B (PAPA/B). Mutations in the *GLI3* gene can also lead to Pallister–Hall syndrome (PHS) and isolated PAPA/B. In this study, we investigated 16 unrelated probands with the clinical diagnosis of GCPS/PPD-IV and found *GLI3* mutations in 12 (75 %) of them (nine familial and three sporadic cases). We also performed a detailed clinical evaluation of all 12 *GLI3*-positive families, with a total of 27 patients. The hallmark triad of GCPS (preaxial polydactyly, macrocephaly/prominent forehead, hypertelorism) was present in 14 cases (52 %), whereas at least one typical dysmorphic feature was manifested in 17 patients (63 %). Upon sequencing of the *GLI3* gene, we demonstrated eight novel and two previously reported heterozygous point mutations. We also performed multiplex ligation-dependent probe amplification (MLPA) to screen for intragenic copy number changes and identified heterozygous deletions in the two remaining cases (16.7 %). Our findings fully support previous genotype–phenotype correlations, showing that exonic deletions, missense mutations, as well as truncating variants localised out of the middle third of the *GLI3* gene result in GCPS/PPD-IV and not PHS. Additionally, our study shows that intragenic *GLI3* deletions may account for a significant proportion of GCPS/PPD-IV causative mutations. Therefore, we propose that MLPA or quantitative polymerase chain reaction (qPCR) should be implemented into routine molecular diagnostic of the *GLI3* gene.

## Introduction

Greig cephalopolysyndactyly syndrome (GCPS, MIM#175700) and isolated preaxial polydactyly type IV (PPD-IV, MIM#174700) are rare autosomal dominant disorders, both caused by mutations in the *GLI3* zinc-finger transcription factor gene (Vortkamp et al. 1991). GCPS is predominantly characterised by craniofacial abnormalities, such as macrocephaly, prominent forehead/frontal bossing, hypertelorism and limb malformation, referred to as PPD-IV, comprising duplicated halluces, with syndactyly of preaxial toes, broad or duplicated thumbs, and syndactyly of the third and fourth fingers. Additionally, postaxial polydactyly type A or B (PAPA/B) may also occur (Temtamy and McKusick 1978). Isolated PPD-IV is often included into the GCPS spectrum, as a subtype in which craniofacial dysmorphic features are mild and indistinguishable from the normal individual (Baraitser et al. 1983). Mutations in the *GLI3* gene can also lead to Pallister–Hall syndrome (PHS) and occasionally to isolated PAPA/B (Kang et al. 1997; Radhakrishna et al. 1997, 1999). Clinical diagnostic criteria for GCPS vary among the studies. Biesceker (2008) proposed a combination of preaxial polydactyly or abnormally broad hallux or thumb in at least one limb, accompanied with syndactyly, macrocephaly and hypertelorism. Since such a full-blown phenotype does not occur in all patients, more relaxed criteria comprising preaxial polydactyly and at least one feature out of syndactyly, macrocephaly and hypertelorism were suggested by Johnston et al. (2005). Characteristic features typical of PHS include the presence of hypothalamic hamartoma, insertional polydactyly and bifid epiglottis (Biesecker 2008).

Two syndromic “GLI3 morphopathies”, GCPS and PHS, are nosologically distinct entities and an efficient algorithm was developed for the prediction of genotype–phenotype correlation. First of all, GCPS is caused by a variety of *GLI3* changes, such as chromosomal rearrangements (translocations, large deletions), exon deletions/duplications, and missense and splicing mutations. Secondly, truncating mutations (frameshift and nonsense) lying between nucleotides (nt) 1–1,997 and nt 3,482–4,740 of the *GLI3* gene result in GCPS, whereas alterations affecting the middle third (nt 1,998–3,481) cause primarily PHS (Johnston et al. 2005, 2010). There is an important biological basis accounting for this genotype–phenotype correlation of truncating variants. For example, mutations located within the amino-terminal third of the protein, which cut off the zinc-finger DNA binding domain, lead to a loss of DNA binding capacity. Additionally, mutations located in the carboxy-terminal third of the GLI3 protein cause a loss of transactivation (TA) domain(s) [TA_1_, amino acids 1,376–1,580; TA_2_, amino acids 1,044–1,322, and CBP-binding module, amino acids 827–1,180] and result in the inability of the protein to activate transcription of the target genes. Conversely, the truncations in the middle part of the gene are predicted to generate constitutive repressors that alter the balance between activator and repressor forms of the GLI3 protein (Ruppert et al. 1990; Dai et al. 1999; Kalff-Suske et al. 1999).

In this report, we present the clinical and molecular data of 27 GCPS/PPD-IV Polish patients from 12 families, all carrying *GLI3* mutations. We extend the mutational spectrum of the gene, assess if the genotype is correlated with the phenotype and discuss the functional consequences of the newly identified GCPS/PPD-IV causative mutations.

## Patients and methods

First, we tested 16 index cases of Polish origin clinically suspected of PPD-IV and/or GCPS and found *GLI3* mutations in 12 of them. Then, from those 12 families for which *GLI3* mutational screening was positive, we recruited 27 patients affected by PPD-IV or GCPS and performed careful clinical investigation. The local ethics committee approved the study and a written informed consent was obtained from all subjects or their legal guardians. Patients were clinically evaluated and X-ray scans of the affected limbs were taken in selected cases. Head circumference was measured and referred to the general population. Macrocephaly was recognised if the parameter exceeded the 97th percentile.

DNA of all index cases was screened for both point mutations and intragenic copy number changes involving the *GLI3* gene, by means of sequencing and multiplex ligation-dependent probe amplification (MLPA). Next, co-segregation testing was performed in all affected and unaffected family members to check for co-occurrence of the detected mutation with the phenotype. Alternatively, parental studies were done in all sporadic cases to confirm a de novo occurrence of the alterations (parental DNA was available for all index patients). The pathogenicity of missense variants was additionally assessed in silico using PolyPhen2 and SIFT software.

Genomic DNA was isolated from whole blood according to the salting-out method. The coding sequence of the *GLI3* gene (GenBank accession number NM_000168.5) comprised 14 exons, and the flanking intronic regions were amplified in polymerase chain reaction (PCR) assays and directly sequenced by means of dye terminator chemistry (kit v.3, ABI 3130XL). Sequences of the primers used for amplification and sequencing PCR reactions are given in Table 1. MLPA for all exons of the *GLI3* gene was performed with the use of a commercial kit P179 per the manufacturer’s protocol (MRC Holland). Data were intra-normalised by dividing the area of each peak by the overall area of the reference probes’ peaks in the probemix. Inter-sample normalisation was obtained by comparing the investigated samples to several reference control samples (healthy individuals) run in the same experiment. Relative peak areas ranging from 0.67 to 1.33 were considered to be normal, below 0.67 deleted and above 1.33 duplicated (Schouten et al. 2002).

**Table 1:** Sequences of the primers used for *GLI3* gene amplification and sequencing

Exon name	F primer sequence 5′–3′	R primer sequence 5′–3′
GLI3_e3	GGCTCTGTTGTTTTCTTTAGGG	GCAAACGCTCAATTCACAAG
GLI3_e4	AGGGATATCGAGAATGAGACC	cacacacacaGCCCTCCC
GLI3_e5	TTGCTTTGTGAATCGGAATG	CCTGAGATGGTAAAAGCCAG
GLI3_e6	CCCAAACAATTGCATAGCG	taacaccactgccaatgagg
GLI3_e7	TGATGTGGGTTGTGTAATGG	GTGGTTCCACTTTCTCCTCC
GLI3_e8	TCTTCCACGTAGGCAAGTAGC	AGGTGCAAACAAGTGCTGAC
GLI3_e9	AACAAATTTGATTTGGGATGG	AGAACACAGAGGTGCCGTG
GLI3_e10	TGGTACTGCTCCTTGTTGATG	CTGACCCAAAGACACCAGTC
GLI3_e11	CCCTCCTGTTGTGTCTGATTC	TCAGCTCAGGGTCAGAGAGG
GLI3_e12	TTTTAAGATTGGGGTATTTTCTGC	TGAGCTGGTGTCATCAGTTTG
GLI3_e13	TTTTCATCAACTTGGAGGGC	CCTTCCCCGGGATAGTTC
GLI3_e14	TAAAGGACTTTTGGGCTGGG	CTATGCACCCTACCTGGCTC
GLI3_e15	ATTGGCTCCCTTTCCTTGAC	ACATAAAACTGAGGGCCTGC
GLI3_e16a	tagttgtgaggcaggcaatg	AGCACGAGACTGCGCTTC
GLI3_e16b	AGTTCATGCCCCGAGGAG	GTAGGGGTTGCTGTTCTCCC
GLI3_e16c	CTCCAAGCTCAAGTGTGGG	ACTGCAGAGCAAGGCTGTC
GLI3_e16d	CGTCAAGCTTGGCAGTTGTC	AAAACAGCCAAAACAAAGTCAG

## Results

### Clinical presentation

#### Family A (four affected members)

**Fig. 1:**
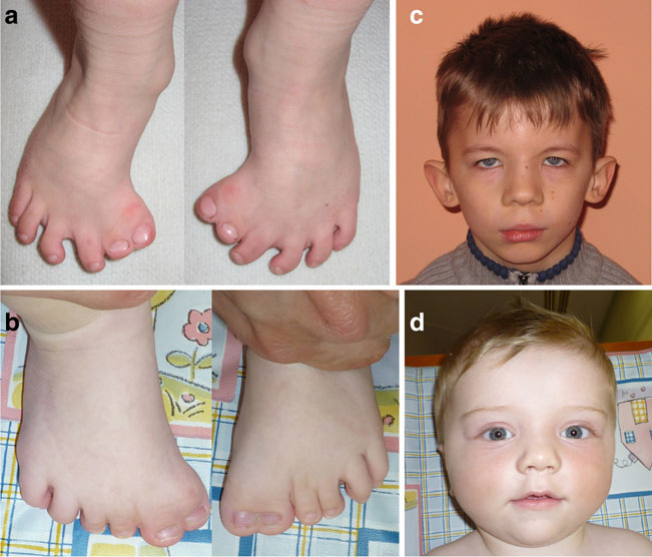
Polysyndactyly of the feet (preaxial polydactyly type IV, PPD-IV) in patients carrying a *GLI3* causative mutation (**a** patient A-2; **b** patient D-1). Facial appearance of the presented *GLI3* mutation carriers; **c**–**d** show craniofacial dysmorphism typical of Greig cephalopolysyndactyly syndrome (GCPS) (**c** patient F-1; **d** patient D-1)

All four patients from this family presented with bilateral preaxial polysyndactyly of the feet and broad thumbs (feet polysyndactyly of patient A-2 is shown in Fig. 1a). Additionally, three patients had bilateral syndactyly of fingers 3/4 (A-1, A-3 and A-4) and clinodactyly (A-2, A-3 and A-4), whereas patient A-2 manifested PAPB. Two patients from this family (A-2 and A-3) had typical craniofacial GCPS features, including macrocephaly and prominent forehead with hypertelorism. Only hypertelorism without macrocephaly/prominent forehead was noted in patient A-1, whereas none of these traits was observed in patient A-4.

#### Family B (two affected members)

Both patients from this family manifested bilateral preaxial polysyndactyly of the feet. In addition, patient B-2 had bilateral hand syndactyly of fingers 3/4 and broad thumbs. Hypertelorism was present in both patients, while only patient B-1 presented with prominent forehead.

#### Family C (sporadic case)

Patient C-1 presented with bilateral preaxial polysyndactyly of the feet, broad thumbs, clinodactyly of the fifth fingers, as well as prominent forehead and hypertelorism.

#### Family D (three affected members)

All three patients from this family presented with bilateral preaxial polysyndactyly of the feet, clinodactyly of the fifth fingers, macrocephaly, prominent forehead and hypertelorism (for foot anomaly and facial dysmorphism of patient D-1, see Fig. 1b, d, respectively). Moreover, patients D-1 and D-2 manifested bilateral syndactyly of fingers 3/4 and broad thumbs.

#### Family E (three affected members)

In two patients from this family (E-1 and E-2), bilateral preaxial polysyndactyly of the feet and PAPB was noted. Broad thumbs were seen in patients E-2 and E-3. Craniofacial GCPS features (i.e. hypertelorism, macrocephaly or prominent forehead) were not present in any of the family members.

#### Family F (sporadic case)

Patient F-1 presented with bilateral preaxial polysyndactyly of the feet, syndactyly of fingers 3/4, as well as craniofacial dysmorphism comprising prominent forehead, hypertelorism, bitemporal narrowing, down-slanted palpebral fissures, epicanthal folds, broad nasal bridge and low-set ears with prominent pinnae (the facial view of patient F-1 is shown in Fig. 1c).

#### Family G (two affected members)

Both patients from this family manifested bilateral preaxial polysyndactyly of the feet, macrocephaly, prominent forehead and hypertelorism. Additionally, patient G-1 had bilateral hand syndactyly of fingers 3/4 and broad thumbs.

#### Family H (two affected members)

Both patients from this family presented with bilateral preaxial polysyndactyly of the feet, as well as broad thumbs. Postaxial polydactyly of the feet, hands (type B) and clinodactyly of the fifth fingers was present only in patient H-1. None of the patients presented with macrocephaly, prominent forehead or hypertelorism.

#### Family I (sporadic case)

Patient I-1 manifested right-sided preaxial foot polysyndactyly, left-sided foot oligodactyly (missing one toe), bilateral PAPA, as well as large head circumference, prominent forehead and hypertelorism.

#### Family J (three affected members)

All three patients from this family presented with bilateral preaxial polysyndactyly of the feet, broad thumbs and bilateral syndactyly of fingers 3/4 (fingers 3/4/5 in patient J-1). Hypertelorism was diagnosed exclusively in patient J-2 and none of the family members manifested macrocephaly.

#### Family K (three affected members)

Preaxial polysyndactyly of the feet (bilateral in patients K-1 and K-2, right-sided in K-3) and broad thumbs were present in all three patients. Hand syndactyly of digits 3/4 was noted in patient K-2. Only patient K-1 presented with typical craniofacial GCPS features (i.e. hypertelorism, macrocephaly and prominent forehead).

#### Family L (two affected members)

Both patients from this family exhibited bilateral preaxial polysyndactyly of the feet, broad and duplicated thumbs, syndactyly of fingers 3/4, macrocephaly, prominent forehead and hypertelorism.

### Molecular findings

Heterozygous *GLI3* mutations were found in 12 out 16 probands with the clinical diagnosis of PPD-IV/GCPS. In three cases, mutation occurred de novo and in nine, it was inherited from an affected parent. To our knowledge, ten alterations were novel and included three missense mutations, four frameshifts, one splicing mutation and two exonic deletions, whereas two (both nonsense variants) were previously reported elsewhere (for a description of the mutations, see Table 2). In all sporadic cases, presence of the mutation was excluded in both healthy parents, thus, confirming their de novo occurrence in the probands. In familial cases, the identified alterations fully co-segregated with the phenotype and were not shown in any of the unaffected family members. In addition, all three missense variants were predicted to be probably damaging to the protein function in the in silico analyses performed by means of both PolyPhen2 and SIFT software.

**Table 2:** Clinical and molecular characteristics of the patients affected by GCPS/PPD-IV

				Clinical phenotype							
				Lower limb		Upper limb				Craniofacial	
Family and patient’s ID (age at evaluation)	Clinical diagnosis	Mutation in the *GLI3* gene	Inheritance (mutation novel/known)	Preaxial polysyndactyly (feet)	Postaxial polydactyly (feet)	Broad or duplicated thumb(s)	Postaxial polydactyly (hands)	Hand syndactyly (fingers 3/4 or 3/4/5)	Clinodactyly	Macrocephaly/prominent forehead	Hypertelorism
A-1 (5 years)	GCPS	c.C3018A (p.S1006R)	F (novel)	+ (bil.)	−	+ (bil.)	−	+ (bil.)	−	−	+
A-2 (3.5 years)	GCPS			+ (bil.)	−	+ (bil.)	+ (B, bil.)	−	+ (bil.)	+	+
A-3 (9 months)	GCPS			+ (bil.)	−	+ (bil.)	−	+ (bil.)	+ (bil.)	+	+
A-4 (33 years)	PPD-IV			+ (bil.)	−	+ (bil.)	−	+ (bil.)	+ (bil.)	−	−
B-1 (3 years)	GCPS	c.C2721G (p.S907R)	F (novel)	+ (bil.)	−	−	−	−	−	+	+
B-2 (28 years)	GCPS			+ (bil.)	−	+ (bil.)	−	+ (bil.)	−	−	+
C-1 (2.5 years)	GCPS	c.G2686A (p.D896N)	S (novel)	+ (bil.)	−	+ (bil.)	−	−	+ (bil.)	+	+
D-1 (31 years)	GCPS	IVS4-2A > G	F (novel)	+ (bil.)	−	+ (bil.)	−	+ (bil.)	+ (bil.)	+	+
D-2 (54 years)	GCPS			+ (bil.)	−	+ (bil.)	−	+ (bil.)	+ (bil.)	+	+
D-3 (6 months)	GCPS			+ (bil.)	−	−	−	−	+ (bil.)	+	+
E-1 (4 years)	PPD-IV	c.C2374T (p.R792X)	F (known)	+ (bil.)	−	−	+ (B, bil.)	−	−	−	−
E-2 (8 years)	PPD-IV			+ (bil.)	−	+ (bil.)	+ (B, bil.)	−	−	−	−
E-3 (33 years)	PPD-IV			−	−	+ (bil.)	−	−	−	−	−
F-1 (10 years)	GCPS	c.497delC(p.P166LfsX50)	S (novel)	+ (bil.)	−	−	−	+ (bil.)	−	+	+
G-1 (8 years)	GCPS	c.1360delC(p.Q454SfsX48)	F (novel)	+ (bil.)	−	+ (bil.)	−	+ (bil.)	−	+	+
G-2 (33 years)	GCPS			+ (bil.)	−	−	−	−	−	+	+
H-1 (5 years)	PPD-IV	c.3383delA(p.D1128AfsX10)	F (novel)	+ (bil.)	+ (bil.)	+ (bil.)	+ (B, bil.)	−	+ (bil.)	−	−
H-2 (29 years)	PPD-IV			+ (bil.)	−	+ (bil.)	−	−	−	−	−
I-1 (2 months)	GCPS	c.4370insGC (p.A1457AfsX32)	S (novel)	+ (R)	−	−	+ (A, bil.)	−	−	+	+
J-1 (19 years)	PPD-IV	exon 4 deletion (in frame)	F (novel)	+ (bil.)	−	+ (bil.)	−	+ (bil.)	−	−	−
J-2 (15 years)	GCPS			+ (bil.)	−	+ (bil.)	−	+ (bil.)	−	−	+
J-3 (37 years)	PPD-IV			+ (bil.)	−	+ (bil.)	−	+ (bil.)	−	−	−
K-1 (5 years)	GCPS	exon 1–9 deletion	F (novel)	+ (bil.)	−	+ (bil.)	−	−	−	+	+
K-2 (8 years)	PPD-IV			+ (bil.)	−	+ (bil.)	−	+ (bil.)	−	−	−
K-3 (28 years)	PPD-IV			+ (R)	−	+ (bil.)	−	−	−	−	−
L-1 (1 month)	GCPS	c.C1096T (p.R366X)	F (known)	+ (bil.)	−	+ (bil.)	−	+ (bil.)	−	+	+
L-2 (25 years)	GCPS			+ (bil.)	−	+ (bil.)	−	+ (bil.)	−	+	+
Total (*n* = 27)				26	1	21	5	14	8	14	17
Σ				26		25				17	

*GCPS* Greig cephalopolysyndactyly syndrome (diagnosed if at least one craniofacial feature out of macrocephaly/prominent forehead and hypertelorism was present)

*PPD-IV* preaxial polydactyly type IV (diagnosed if neither macrocephaly/prominent forehead nor hypertelorism was present)

*F* familial; *S* sporadic

(+) = feature observed in individual; (−) = feature not seen in individual

*R* right-sided; *L* left-sided; bil. = bilateral

*A* refers to type A postaxial polydactyly (fully formed digit); *B* refers to type B postaxial polydactyly (hypoplastic or rudimentary digit)

In total, we studied 27 patients exhibiting either GCPS or isolated PPD-IV phenotype, in whom causative *GLI3* mutations were detected. Twenty-six patients presented with typical lower limb malformation (preaxial polydactyly and syndactyly of preaxial toes, Fig. 1a, b), whereas 25 had at least one of the following hand affectations (broad thumb, syndactyly fingers 3/4 or 3/4/5, clinodactyly, PAPA/B). Detailed clinical characteristics of the presented cohort is set out in Table 2, whereas facial dysmorphism seen in some of our GCPS patients is shown in Fig. 1c, d. The hallmark triad of GCPS (preaxial polydactyly, macrocephaly and hypertelorism) was present in 14 cases (52 %). At least one typical craniofacial GCPS feature (macrocephaly/prominent forehead or hypertelorism) was manifested in 17 patients (63 %). In total, ten cases presented with no facial dysmorphism, thus, PPD-IV seemed to be the most appropriate diagnosis.

## Discussion

*GLI3* mutations are associated with several human syndromic [GCPS, PHS, acrocallosal syndrome] and non-syndromic (isolated) congenital limb malformations [PPD-IV, PAPA/B] (Vortkamp et al. 1991; Kang et al. 1997; Radhakrishna et al. 1997, 1999; Elson et al. 2002). While clinical criteria for PHS are clear and require the presence of a hypothalamic hamartoma and insertional polydactyly or isolated hamartoma or polydactyly in a relative of the proband with PHS (Biesecker 2006, 2008), diagnostic criteria for GCPS are neither fully defined nor widely accepted. Following the strict diagnostic criteria provided by Biesceker (2008), which included preaxial polydactyly/wide big toes or thumbs with syndactyly, macrocephaly and hypertelorism, we would be able to recognise the syndrome in 14 out of 27 mutation carriers from the presented cohort. If either macrocephaly/prominent forehead or hypertelorism was considered as a sufficient craniofacial trait, GCPS could be recognised in only 17 patients. No facial characteristics, a key feature of GCPS, was observed in ten patients, suggesting that isolated PPD-IV would be the most appropriate diagnosis in this case.

In 2005, Johnston et al. (2005) proposed relaxed criteria unhelpful in making the GCPS diagnosis, but useful in selecting patients for *GLI3* molecular analysis. The authors suggested that patients presenting with preaxial polydactyly and at least one of the following features, syndactyly, macrocephaly or hypertelorism, would benefit from the *GLI3* testing. Of note, out of our 16 unrelated probands analysed in this study, 12 (i.e. 75 %) showed a positive result of *GLI3* screening, thereby, confirming the relatively high efficacy of the aforementioned criteria in predicting an abnormal molecular result.

**Fig. 2:**
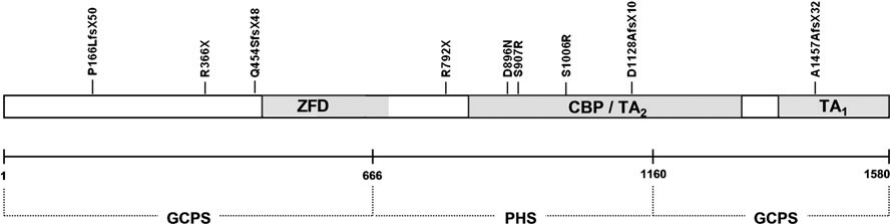
Schematic view of the *GLI3* gene structure and overview of all exonic point mutations identified in this study. ZFD, zinc-finger domain [amino acids (aa) 462–645] reported by Ruppert et al. (1990). CBP, CBP-binding domain (aa 827–1,132) reported by Dai et al. (1999). TA_2_ transactivation domain (aa 1,044–1,322) reported by Kalff-Suske et al. (1999). TA_1_ transactivation domain (aa 1,376–1,580) reported by Kalff-Suske et al. (1999). GCPS, Greig cephalopolysyndactyly syndrome. GCPS is caused by truncating mutations lying between aa 1–666 and 1,160–1,580 of the protein (Johnston et al. 2005, 2010). PHS, Pallister–Hall syndrome. PHS is caused by mutations affecting the middle third (aa 667–1,160) of the protein (Johnston et al. 2005, 2010)

Among all mutations identified in this study, the majority were truncating variants, including four novel frameshift mutations, two previously reported nonsense mutations and two exonic deletions. Frameshifts at codon 166 (p.P166LfsX50; proband F-1) and 454 (p.Q454SfsX48; family G), as well as a nonsense mutation (p.R366X; family L) and an out-of-frame exon 4 deletion (family J), introduce a premature stop codon, which cause the truncation of the GLI3 protein synthesis within its N-terminal portion. Thereby, the mutant proteins lack all functionally important domains, including a highly conserved zinc-finger domain (ZFD), which is crucial for DNA binding capacity in a sequence-specific manner (Kinzler and Vogelstein 1990). A similar pathogenic mechanism (i.e. loss of DNA binding potential) is predicated to take place in case of a splicing mutation detected in family D, in which we identified A to G substitution in the AG sequence of the acceptor site at position −1 of intron 4. This mutation is supposed to result in exon skipping and premature truncation of the protein synthesis. The other two detected frameshifts at codon 1,128 (p.D1128AfsX10; family H) and 1,457 (p.A1457AfsX32; proband I) truncate the GLI3 protein C-terminally to the DNA binding domain, and localise within the transactivation domains TA_2_ and TA_1_, respectively. The nonsense mutation p.R792X (family E) terminates the protein downstream to both TA domains. Mutations affecting those regions most probably result in misregulation of the *GLI3*-mediated transcriptional activation of the target genes (Kalff-Suske et al. 1999). Another mutation identified in family K, a heterozygous intragenic deletion of exons 1–9, most probably results in a complete loss of one gene copy (haploinsufficiency). Interestingly, all three missense mutations (p.S1006R, family A; p.S907R, family B; p.D896N, proband C) mapped within the CBP-binding module. All three missense variants were predicted to be probably damaging to the protein function in analyses by means of both PolyPhen2 and SIFT software. An overview of all identified mutations, along with their intragenic location and putative pathogenic mechanism, is presented in Table 3 and Fig. 2.

**Table 3:** Location of the mutations within *GLI3* domains and putative pathogenic mechanism of each mutation. Pathogenicity of missense variants was additionally assessed by PolyPhen2 and SIFT software

Family ID	Clinical phenotype	Mutation in the *GLI3* gene	SIFT score	PolyPhen2 score	Domain	Putative pathogenic effect
A	GCPS/PPD-IV	c.C3018A (p.S1006R)	0	1	CBP/TA	Loss of transactivation potential
B	GCPS	c.C2721G (p.S907R)	0	1	CBP/TA	Loss of transactivation potential
C	GCPS	c.G2686A (p.D896N)	0.01	1	CBP/TA	Loss of transactivation potential
D	GCPS	IVS4-2A > G	N/A	N/A	N/A	Loss of the DNA-binding capacity
E	PPD-IV	c.C2374T (p.R792X)	N/A	N/A	X N-terminal to TA_2_, TA_1_	Loss of transactivation potential
F	GCPS	c.497delC (p.P166LfsX50)	N/A	N/A	X N-terminal to ZFD	Loss of the DNA-binding capacity
G	GCPS	c.1360delC (p.Q454SfsX48)	N/A	N/A	X within ZFD	Loss of the DNA-binding capacity
H	PPD-IV	c.3383delA (p.D1128AfsX10)	N/A	N/A	X within TA_2_	Loss of transactivation potential
I	GCPS	c.4370insGC (p.A1457AfsX32)	N/A	N/A	X within TA_1_	Loss of transactivation potential
J	GCPS/PPD-IV	exon 4 deletion (out-of-frame)	N/A	N/A	X N-terminal to ZFD	Loss of the DNA-binding capacity
K	GCPS/PPD-IV	exon 1–9 deletion	N/A	N/A	N/A	Haploinsufficiency
L	GCPS	c.C1096T (p.R366X)	N/A	N/A	X N-terminal to ZFD	Loss of the DNA-binding capacity

*GCPS* Greig cephalopolysyndactyly (diagnosed if at least one craniofacial feature out of macrocephaly/prominent forehead and hypertelorism was present)

*PPD-IV* preaxial polydactyly type IV (diagnosed if neither macrocephaly/prominent forehead nor hypertelorism was present)

*N/A* not applicable

*GCPS/PPD-IV* coincidence of both phenotypes within the same family

*CBP/TA* CBP-binding domain

*X* stop codon

*TA*_*2*_, *TA*_*1*_ transactivation domains

*ZFD* zinc-finger domain

SIFT score: the amino acid substitution is predicted to be damaging if the score is ≤0.05 and tolerated if the score is >0.05

PolyPhen2 score: the amino acid substitution is predicted to be damaging if the score is above 0.85

According to the genotype–phenotype correlation, GCPS, unlike PHS, can be caused by different alterations of the *GLI3* gene, including chromosomal rearrangements (translocations, large deletions), exon deletions/duplications, and missense and splicing mutations. Among truncating mutations (i.e. frameshift and nonsense), those lying in the N-terminal and C-terminal third of the protein result in GCPS, whereas alterations affecting the middle third cause PHS (Johnston et al. 2005, 2010). The type and position of the novel mutations detected in our patients showed full correlation between the expected and observed phenotype. The only exception was a recurrent mutation c.C2374T (p.R792X) identified in one of our families, localised in the PHS region. This variant was previously described in six families presenting with the GCPS phenotype (Kalff-Suske et al. 1999; Johnston et al. 2005). In our case, a p.R792X mutation was responsible for isolated PPD-IV, since none of the three affected members (E-1 to E-3) had craniofacial involvement. To conclude, our findings fully support previous genotype–phenotype correlations corroborating the usefulness and high predictive value of the algorithm published by Johnston et al. (2005). On the other hand, one has to emphasise that any genotype–phenotype correlations based on single mutation carriers or families are, at best, tentative. Even within the same family, in which the molecular cause of the disease is identical, there might be a significant variability of the clinical symptoms between the affected kindreds (Cohen 2010). Therefore, further studies are needed in order to prove whether the correlations for the mutations presented within this report are correct.

In 2 out of our 12 probands (16.7 %), MLPA demonstrated heterozygous intragenic causative deletions. This type of mutation was recently associated with metopic and/or sagittal synostosis (Hurst et al. 2011); however, none of our 6 patients (J-1 to J-3, K-1 to J-3) who carried the deletion had anomalies of the cranial sutures. Although based on a small sample, our study shows that intragenic *GLI3* deletions may account for a significant proportion of GCPS/PPD-IV causative mutations. Therefore, we propose that MLPA or quantitative polymerase chain reaction (qPCR) should be implemented into routine molecular diagnostic of the *GLI3* gene, especially if the sequence analysis detects no pathogenic alteration.
